# Acral Lentiginous Melanoma *in Situ*: A Diagnostic and Management Challenge 

**DOI:** 10.3390/cancers2020642

**Published:** 2010-04-20

**Authors:** Hyun Sun Park, Kwang Hyun Cho

**Affiliations:** Department of Dermatology, Seoul National University College of Medicine, 28 Yongon-dong, Chongno-gu, Seoul 110-744, Korea; E-Mail: snudm@paran.com

**Keywords:** acral lentiginous melanoma, acral lentiginous melanoma *in situ*, acral melanoma *in situ*

## Abstract

Early stage recognition of acral lentiginous melanoma (ALM) is important for a better prognosis, but in-depth understanding and proper management of ALM *in situ* is complicated, because there are only a few reports, probably due to its rarity and diagnostic difficulty. We have reviewed our experience with seven patients who were diagnosed as having ALM *in situ* and discuss how to accurately diagnose and properly manage these rare lesions. Clinically the lesions showed black to brown discoloration of the nail with Hutchinson’s sign and hyperpigmented macules on the heel with color variegation. All the lesions showed a diffuse lentiginous pattern of melanocytic proliferation with variable level of atypism along the dermoepidermal junction. Dermoscopic findings were available in three and revealed parallel ridge patterns. Confrontation of clinical and histopathologic findings was observed in three, and the lesions were not recognized or diagnosed as ALM *in situ* in the first place. Excision of the primary lesion with variable operative margin was done as an initial treatment. Recurrence was observed in three patients and one developed invasive ALM and lymph node metastasis. Integration of all available information concerning the clinical presentation, histopathology, and dermoscopic findings is very important and can lead to the best classification for correct diagnosis. Lack of knowledge upon clinical course and optimal margin to control ALM *in situ* provokes the need for further studies with longer follow up and larger number of cases.

## 1. Introduction

Since it was first reported by Reed [1] in 1976, acral lentiginous melanoma (ALM) has been regarded as a rare but distinctive subtype of melanoma. ALM was considered more aggressive than other subtypes [2,3] and it was unusual to encounter patients showing only the histopathologic features of its radial growth phase, in other words, ALM *in situ*. However, recent reports [4,5] suggest that this was due to late detection or delayed diagnosis, rather than a true biological difference. Therefore, early diagnosis of ALM is important for a better prognosis but its diagnosis is not always easy and it is often misdiagnosed. There are only a few reports upon ALM *in situ*, probably due to its rarity and diagnostic difficulty, which makes in depth understanding and proper management of ALM *in situ* complicated. Therefore, we have reviewed our experience with ALM *in situ* and discussed how to accurately diagnose and properly manage these rare lesions.

## 2. Results and Discussion

### 2.1. Clinical Features

**Figure 1:**
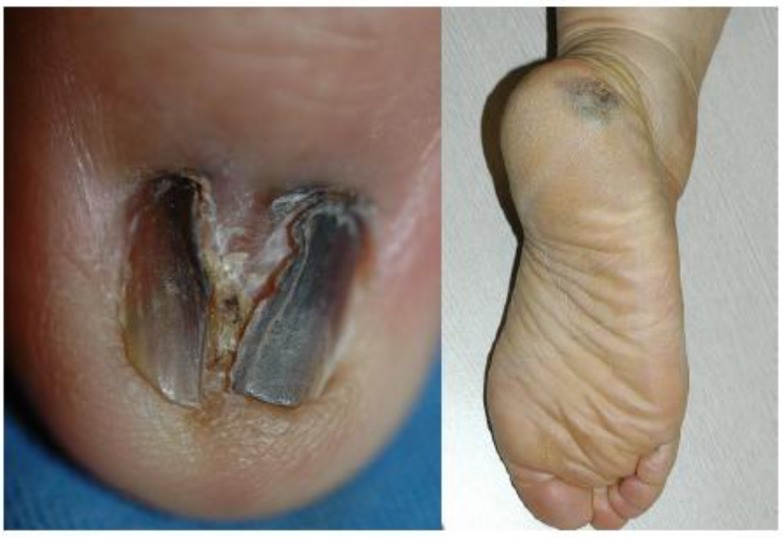
(A) Patient 4: black to brown colored pigmented patch on the left ring finger (B) Patient 7: irregularly pigmented patch on the right heel.

The clinical features and information about treatment outcome are summarized in Table 1. There were seven patients with acral melanoma *in situ* and the male to female sex ratio was 3 to 4. They were all Korean and aged 47–75 years with a mean age of 58.4 years (median, 58.0 years) at diagnosis. The average duration before presentation was 3.0 years (range, 1-5; median, 3.0 years). All the patients complained of a pre-existing lesion which is increasing in size or spreading to adjacent area. Four lesions occurred on the heel and three on the fingernail. Most of the lesions located on the nail apparatus showed black to brown discoloration involving the whole nail plate, nail deformation, and Hutchinson’s sign at the time of diagnosis (Figure 1A), whereas patient 6 initially presented a longitudinal black streak increasing in width and Hutchinson’s sign. 

The lesions on the heel (Figure 1B) exhibited hyperpigmented macules with color variegation and their maximum diameter ranged from 0.6 to 4.0 cm (average, 1.95; median 1.6). Records of dermoscopic findings were available in three lesions on the heel (patients 2, 5 and 7), and all of them revealed parallel ridge patterns.

**Table 1:** Summary of the patients.

Patient number	Age/sex	Site	Duration (years)	Maximum diameter (cm)	Initial treatment	Follow up period (months)	Recurrence	Clinical status
1	46/F	heel	unknown	0.7	5 mm margin excision, clear resection margin	39	no recurrence	alive without evidence of disease
2	58/F	heel	1	0.6	3 mm margin excision, clear resection margin	51	after 24 months, local recurrence	alive after recurrence
3	47/M	finger nail, 5th	5		excisional biopsy, involvement of resection margin	120	after 84 and 96 months, local recurrence and nodal metastasis	alive without evidence of disease after amputation (clear resection margin), chemotherapy and lymph node dissection
4	50/F	finger nail, 1st	3		amputation, clear resection margin	32	no recurrence	alive without evidence of disease
5	75/M	heel	5	2.5	1cm margin excision, clear resection margin	9	no recurrence	alive without evidence of disease
6	74/M	finger nail, 4th	3		excisional biopsy and nail extraction, no information upon resection margin	144	after 120 months, local recurrence	alive without evidence of disease after 5 mm margin excision (clear resection margin)
7	59/F	heel	3	4.0	1cm margin excision, clear resection margin	15	no recurrence	alive without evidence of disease

### 2.2. Histopathologic Features

**Figure 2:**
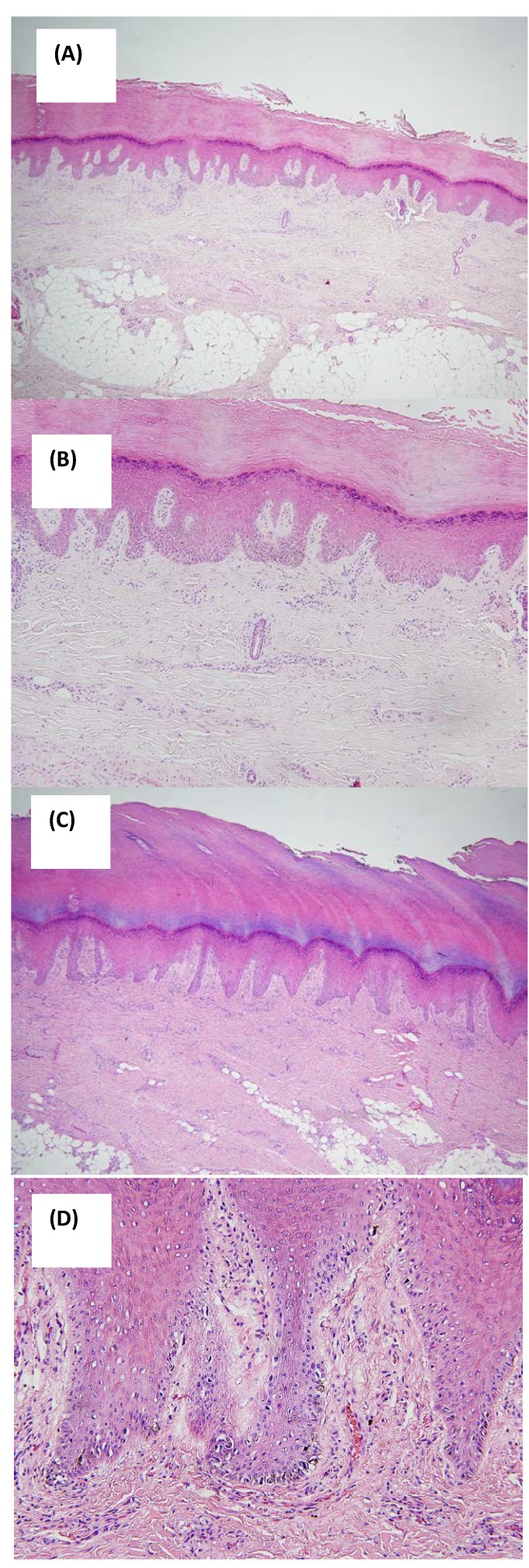
(A) Patient 5: retiform epidermal hyperplasia with somewhat broad rete pegs (H&E, ×40); (B) Patient 5: diffuse lentiginous pattern of melanocytic proliferation along the dermo-epidermal junction with hyperplastic epidermis compatible with ALM *in situ* (H&E, ×100); (C) Patient 7: irregular epidermal hyperplasia with compact hyperkeratosis (H&E, ×40); (D) Patient 7: large atypical melanocytes with irregular shapes and hyperchoramatic nuclei (H&E, ×200).

All seven lesions showed a diffuse lentiginous pattern of melanocytic proliferation along the dermoepidermal junction with hyperplastic epidermis (Figure 2A–D). Two cases (patient 2 and 6) showed melanocytic hyperplasia with focal atypism but most cases revealed diffuse large atypical melanocytes with irregular shapes and hyperchromatic nuclei (Figure 2D). Junctional nest formation was not observed. The melanocytes were localized to basal layers in three cases (patients 2, 6 and 7) whereas some melanocytes were also present in the spinous layers in the others. In three cases where dermoscopic findings were available, prominent intraepidermal proliferation of melanocytes in the crista profunda intermedia corresponding to the dermoscopic features of parallel ridge pattern was observed only in some areas. Immunohistochemical study was performed in six cases. All the lesions were positively stained with anti-HMB-45 but only weakly with anti-S-100 antibodies. 

### 2.3. Diagnosis

The interval between the first detection by the patient and the diagnosis of ALM *in situ* ranged from 1 to 13 years. Confrontation of clinical and histopathologic findings was observed in three (patients 2, 3 and 6), and the lesions were not recognized or diagnosed as ALM *in situ* in the first place. Patient 2 showed clinically ambiguous pigmented lesion with relatively small diameter and symmetrical structure. But based upon parallel ridge pattern on dermoscopy indicating malignant melanoma, excisional biopsy was performed. The patient was finally diagnosed as early ALM *in situ* after integrating clinical, histopathologic, and dermoscopic findings. In patient 3, clinical findings suggested melanoma. Lateral excisional biopsy was initially done, but it did not reflect the whole structure. The specimen revealed subtle changes insufficient to be diagnosed as melanoma. However, total excision afterward demonstrated histopathological findings compatible with ALM *in situ*. Patient 6 presented with seemingly malignant but histopathologically ambiguous lesion showing proliferation of melanocytes with inconspicuous atypism. It was initially diagnosed as a non-malignant lesion, but the clinical evolution and recurrence of histopathologically overt ALM *in situ* supported the diagnosis of early ALM *in situ*.

### 2.4. Treatment and Outcome

Excision of the primary lesion with variable operative margin was done as an initial treatment. Misdiagnosis or delayed diagnosis led to inappropriate treatment. Two had excisional biopsy only with or without nail extraction and one had 3 mm margin excision. In the others, three had wide excision and skin graft (one with 5mm margin and two with 1 cm margin), and one had amputation. 

Follow-up period ranged between 9 and 144 months with a mean time of 59.1 months (median, 39 months). Extended follow-up of more than 1 year was available in 6 of 7 patients. Recurrence was observed in three patients during the follow-up period. In patient 2, early ALM *in situ* recurred on the same area 24 months after 3 mm margin excision. Patient 3 developed invasive ALM on the primary excision scar 7 years after excisional biopsy. One year thereafter, left axillary lymph node enlargement was detected by fusion positron emission tomography. Nodal metastasis was confirmed after lymph node dissection and the patient was treated with adjuvant multi-agent chemotherapy (six cycles of cisplatin, vinblastine and dexamethasone). In patient 6, ALM *in situ* recurred on the previous excision scar 10 years after excisional biopsy. The lesion was treated with 5 mm margin. The state of patients at the last contact was investigated. Death related to melanoma did not occur and most patients were alive without evidence of disease except for one.

### 2.5. Discussion

ALM occurs on the hairless skin of palms, soles or beneath the nail plate. Clinically it shows a brown to black macule or patch with color variation and irregular borders. Histopathologicially, it is characterized by the lentiginous proliferation of radial growth phase. In white skinned-population, ALM is rare and accounts for approximately 5% of all melanomas [6] and subungual variant is even rarer [3]. In Asians including Korean, melanoma most commonly involves acral parts and ALM is the most common histopathological subtype [7,8]. But ALM is still rare in Asians like in white-skinned population because the overall incidence of melanoma is very low. Although ALM shares some histopathologic findings with lentigo maligna melanoma, it has been considered more aggressive with worse prognosis [2,3]. However, recent studies [4,5] suggest that its worse prognosis may result from late detection or delayed diagnosis rather than a true difference in biologic nature. Therefore, it is important for clinicians to accurately diagnose and properly manage ALM *in situ* for a better prognosis.

However, previous guidelines for improved recognition of suspicious pigmented lesions are not useful for those on the acral area. For example, the well-known ABCD rule cannot be applied to the pigmented lesions on the palms, soles or nail units due to peculiar anatomy and continuous outgrowth of the nail plate [9]. Therefore, Saida *et al.* [10] reviewed pigmented lesions on the plantar area including melanocytic nevus, dysplastic nevus, malignant melanoma *in situ* and malignant melanoma, and suggested that maximum diameter of a lesion is the only objective discriminating factor. Then he proposed that pigmented lesions on the sole more than 7 mm in diameter should be resected and examined histopatholgocially. And in subungual variant, the spread of pigment into the proximal or lateral nail folds known as Hutchinson's sign is strongly suggestive of subungual melanoma [11] and calls for histopathological examination. 

Histopathological examination is very helpful and essential for the diagnosis of ALM. But in ALM *in situ*, especially in an early stage, the histopathological changes are often equivocal or does not correspond to clincial findings, which makes the accurate diagnosis extremelely difficult. In these cases, dermoscopy can give additional information. The dermoscopic patterns of acral area have been well studied in Asians and Saida *et al.* [12] reported that the parallel ridge pattern showed high positive predictive value and diagnostic accuracy for melanoma *in situ* on acral volar skin. Oguichi and co-workers [13] also reported that parallel ridge pattern showed very high specificity and sensitivity for melanoma *in situ* on glabrous skin. Furthermore, some authors also reported that dermoscopy can identify early acral melanoma *in situ* before they are diagnosed by conventional clinical or histopathological criteria [14,15,16]. Recently, dermoscopic findings of ALM of nail apparatus were also analyzed [9,17]. These studies reported that an irregular lines pattern is the most prominent dermoscopic feature of ungual ALM, and triangular shape with a larger proximal than distal edge of the longitudinal band was observed in ungual ALM *in situ*. Confrontation between clincial and histopathologic findings is also observed when a specimen is incompletely excised, causing midiagnosis or delayed diagnosis. Therefore, when clinical and histopathological findings do not concur, consideration should always be given to performing a further biopsy [18]. Furthermore, histopathologic findings shows only a still image of the entire biologic process, they are somtimes misleading especially in slowly progressing or early lesions including ALM *in situ*. In these cases, close clinical or dermoscopic follow up can give information about the evolution of the lesions. 

The primary treatment of ALM *in situ* is surgical excision but there had been much debate on the issue of surgical margin. The international consensus was that 1cm margin is appropriate in melanoma *in situ*. Later, the National Institues of Health Melanoma Consensus panel suggested that 5 mm margin is effective. Although narrow margins as little as 3 mm were also recommended, subsequent studies using Mohs surgery have shown that these may not be adequate [19,20]. However, Zitelli *et al.* [20] suggested that melanoma of acral areas may require wider surgical margin than anticipated and recommended minimum margin of 1.5 cm. In cases where such large margins can compromise the cosmetic or functional outcome, alternative surgical techniques like Mohs surgery may be considered and can give successful results [21,22]. However, the recent survey upon management of melanoma *in situ* [23] shows that it is still highly controversial as to the ideal surgical margin of melanoma *in situ* including ALM *in situ*. The respondents were divided between supporting a surgical margin of 5 mm or less (54.8%) and greater than 5 mm (33.3%) for melanoma *in situ* not on the face. Our limited experience demonstrates that 3 mm margin seems inappropriate for ALM *in situ* but 5 mm or 1 cm margin may give promising results. ALM *in situ* presents challenges to surgeons and it is clear that large scale prospective controlled research with long term follow up to demermine recurrence rates and survival is required to examine the optimal margin for AML *in situ*.

It is considerably uncertain about the natural course of ALM *in situ*. There are no large scale studies but only several anecdotal cases revelaing malignant transformation or clinical course of ALM *in situ*. A case was recently reported of ALM *in situ* which had slowly progressed to invasive ALM over 12 years [24]. In a case series of ALM *in situ* by Kwon *et al.* [4], the duration of ALM *in situ* ranged from 5 to 30 years and none progressed to invasive melanoma for the follow up period. In our study, most patients had skin lesions for several years before they visited us, and they showed a rather indolent course. However, patient 3 developed recurrence of invasive ALM and lymph node metastasis 12 and 13 years after the first onset of ALM *in situ*, respectively. We believe ALM *in situ* form a disease spectrum where some are extremely indolent over decades and a minority may develop invasive melanoma in the end. It seems hard to predict from the beginning which one will progress to invasive melanoma and it provokes the need for close observation and further studies with longer follow up and larger number of cases. 

## 3. Experimental

We searched for patients who were followed up for melanoma between 2001 and 2008 through electronic medical records and histopathologic database of the Departments of Dermatology and Pathology, Seoul National University College of Medicine, Seoul, Korea. The search found 75 patients from database and 45 patients showed melanomas on the acral area with an intraepidermal component that satisfies the definition of ALM. Among these patients, seven patients were diagnosed as having ALM *in situ* after systematic review and they were finally included in this study. 

### 3.1. Clinical Findings

Retrospective review of medical records and clinical photographs was performed and the following clinical data were retrieved: age at diagnosis, sex, race, duration of the lesion before diagnosis, site of the lesion, description of the primary lesion, maximaum diameter, initial diagnosis and presence or absence of Hutchinson’s sign for the subungual melanomas. Whenever available, dermoscopic photographs obtained by an epiluminiscence microscopy (Dermlite FOTO, CA, USA) were examined or medical records were reviewed for the following dermoscopic features including parallel ridge pattern, parallel furrow or latticelike pattern, fibrillar pattern, irregular dots/globules, abrupt edge and irregular diffuse pigmentation. For each patient, information on treatment and follow-up was investigated: presence or absence of recurrence, date of recurrence, type of recurrence, and clinical status at the latest contact. 

### 3.2. Histopathology

Hematoxylin and eosin stained sections of paraffin-embedded, formalin-fixed skin tissue were examined by light microscopy. The diagnosis of acral melanoma *in situ* was confirmed when the lesion showed proliferation of atypical melanocytes with large irregular and hyperchromatic nuclei in a lentiginous pattern along the dermoepidermal junction without dermal invasion. The sections were reviewed for absence or presence of nest formation, hyperplastic epidermis, and a distribution of atypical melanocytes. 

### 3.3. Immunohistochemistry

Immunohistochemical staining was done using a labeled streptavidin-biotin peroxidase complex method with DAKO LSAB kit (DAKO Korea, Seoul, Korea), similar to those previously described [25]. Primary antibodies used were anti-S-100 (DAKO Korea, Seoul, Korea) and anti-HMB-45 (DAKO Korea, Seoul, Korea).

## 4. Conclusions

We reviewed our experience with ALM *in situ*. It is a diagnostic challenge and integration of clinical-histopathologic-dermoscopic findings is very important for the accurate diagnosis or early detection of ALM *in situ*. When diagnosing the case with confrontaion between clinical and histopathologic findings, we have to be careful and if necessary, consider further complete biopsy. Lack of knowledge upon clinical course and optimal margin to control the disease presents a management challenge and provokes the need for further studies with longer follow up and larger number of cases. 
